# Applications of Intelligent Model to Analyze the Green Finance for Environmental Development in the Context of Artificial Intelligence

**DOI:** 10.1155/2022/2977824

**Published:** 2022-07-07

**Authors:** D. Hemanand, Nilamadhab Mishra, G. Premalatha, Dinesh Mavaluru, Amit Vajpayee, Sumit Kushwaha, Kibebe Sahile

**Affiliations:** ^1^Department of Computer Science and Engineering, S. A. Engineering College (Autonomous), Poonamallee-Avadi Main Road, Veeraragavapuram, Thiruverkadu, Chennai, India; ^2^School of Computing Science and Engineering, VIT Bhopal University, Bhopal, Madhya Pradesh, India; ^3^Department of Electronics and Communication Engineering, Prathyusha Engineering College, Thiruvallur 602025, Tamilnadu, India; ^4^Department of Information Technology, College of Computing and Informatics, Saudi Electronic University, Riyadh, Saudi Arabia; ^5^Chitkara University Institute of Engineering and Technology, Chitkara University, Rajpura, Punjab, India; ^6^Department of Computer Applications, University Institute of Computing, Chandigarh University, Mohali, India; ^7^Department of Chemical Engineering, College of Biological and Chemical Engineering, Addis Ababa Science and Technology University, Addis Ababa, Ethiopia

## Abstract

Green finance can be referred to as financial investments made on sustainable projects and policies that focus on a sustainable economy. The procedures include promoting renewable energy sources, energy efficiency, water sanitation, industrial pollution control, transportation pollution control, reduction of deforestation, and carbon emissions, etc. Mainly, these green finance initiatives are carried out by private and public agents like business organizations, banks, international organizations, government organizations, etc. Green finance provides a financial solution to create a positive impact on society and leads to environmental development. In the age of artificial intelligence, all industries adopt AI technologies. In this research, we see the applications of the intelligent model to examine the green finance for ecological advancement with regard to artificial intelligence. Feasible transportation and energy proficiency and power transmission are two significant fields to be advanced and focused on minimizing the carbon impression in these industries. Renewable sources like solar energies for power generation and electric vehicles are to be researched and developed. This R&D requires a considerable fund supply, thus comes the green finance. Globally, green finance plays a vital role in creating a sustainable environment. In this research, for performing the green finance analysis, financial maximally filtered graph (FMFG) algorithm is implemented in different domains. The proposed algorithm is compared with the neural model and observed that the proposed model has obtained 98.85% of accuracy which is higher than the neural model.

## 1. Introduction

In 2021 the Chinese government issued a rule related to the environment's growth by facilitating the efforts toward green finance. Green credit policy (GCP) also provides enough incentives to high enterprises by focusing on it through their investments [1]. In this paper [2], the author used to explain the difference between the cluster mechanism and the development of human cognition. There are no prior artificial parameters; according to the strategy, the algorithms have been defined in such a case. Environmental development should always be concerned in all growth sets as the author [3] raised an economic possibility towards the rule in the request to raise financial support to overcome the issues faced by the wind electricity sector and the power sector. The author also explains the necessity to reduce fossil fuel consumption by increasing wind power and solar production availability. One of the essential things to hold on to economic growth is that there would be a rise in environmental challenges. Even though there are different managing sectors to develop our ecological maintenance and separate taxes in advance, these are being addressed by the failure of markets and other environmental impacts [4].

### 1.1. Contribution

The main contribution of the work is to develop an environment by implementing a green finance procedure. The allocation can be provided for the maintenance of natural resources, ecology, and human development and also to share information in some cases. It also focuses on the reduction of human intervention in natural disasters or calamities. Among the transportation, healthcare needs, industrial sector, and the persons in need of financial management.

## 2. Literature Review

The development of green finance is always considered to play a pivotal role that helps accelerate the complexity and uncertainty to form a guaranteed relationship between the green economy developments and its traditional networks [5]. There would be some relation between the environmental developments and one of the top multinational companies. Through this article [6], the author explains the change that should be made through the multinational companies, even in such companies' financial decision entity might have integration and that would help through reflecting the costs by the present accounting period. Recognition made from the circular economy has some practical applications to manage those environmental and economic growths. This kind of digital transformation has been implemented through the fourth industrial development related to circular economic aspects (CE). Two other questions have been answered by the author [7] related to the support of intellectual assets and comparing the initiatives. In China, their main prospect is to improve their technologies, which would create a low-carbon economy. This decision has been taken after facing the financial crisis that occurred globally. There would be a situation designed to manage a separate financial institution model or the green environmental model by low carbon production [8]. While here [9], the author focused on promoting the modern green method by improving the green credit risk management, industrial banks also have a significant part in developing the environmental risks. This aids in reducing the effects on such commercial banks. The only chance is to improve green credit risk management.

In this article [10], the author had to explain the development of a real-coded genetic algorithm (RCGA) which helps in the field of filtration and modeling on the basis of finding accurate diversities. After completing this experiment, the results have been adapted to the real-world applications of the CARS. This paper [11] brought us the idea of environmental development and a few of its aspects that help convey the concept in all such cases. If there is a connection between cloud computing and artificial intelligence, getting additional features might be easier with a particular reference. Multicontext systems usually diagnose the ranking system even if it does not require other knowledge. The author has explained the exchange of direct exchange under different perspectives [12]. Delegation is a set of authorized processes that vary under a constant device from one user to another. The transfer of such a type function deals with the importance of delegation by implementing those basic security policies. Explaining the characteristics of the delegation would make the concept a bit more interesting and be used for reformalization [13]. In this article [14], the author has given the deep learning concept of how the ontology is being used in real-time applications using its model approach. However, the linking context makes the research develop automatic interpretation and other capabilities. Globally, there are a lot of globally affected climatic conditions. It causes a significant impact on people in some cases if on one hand nature is the reason for such climatic changes and on the other hand, humans are the cause . Through this article [15], the author has explained the relationship and recovery of artificial intelligence and cloud computing.

## 3. Proposed Model

**Figure 1:**
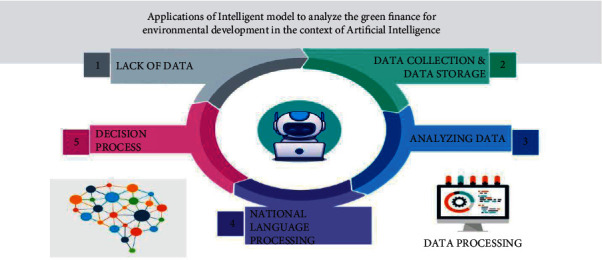
Proposed model of the green finance.

In green finance, there is a lack of data on the client companies who carry out R&D on sustainable energy projects. To predict a company's growth, sustainable finance firms and banks have to access the company's environmental, social, and governance (ESG) data. The process involved in the application of the intelligent model to analyze the green finance for environmental development in the context of AI is defined in Figure 1, and the methods are as follows:

1. Lack of data: in green finance, lack of data is a significant drawback previously. In the age of internet technology, there is no shortage of data. But, the reliability of the data available has to be checked and analyzed before taking it as input data. Data is collected from open sources and analyzed for its reliability, thus labeling it as valuable data. Data plays a vital role in green finance. Collection of data and processing of the collected data is essential.
2. Data collection and data storage: a company's sustainable growth is predicted with the help of its ESG data that is available in open source. The environmental, social, and governance (ESG) data of the company available in open source and is collected and stored. The collection and storage of data is a necessary process. Once the data is collected and stored, the AI takes care of the rest of the work involved. Artificial Intelligence (AI) compares the data from various sources about a particular client and analyzes it.
3. Analyzing data: the data collected and stored is interpreted by the artificially intelligent model by comparing all the data provided for one particular client. The ESG data is of great importance in this process. The AI helps the banks store all the valuable data available on the client, and an ESG Score is given for that client. This is used for further proceedings of the transaction.
4. Natural language processing: the Natural Language processing (NLP) is an AI technology that understands human languages. It performs various tasks such as translation, data classification on topics, etc., at ease. The NLP is used to analyze the Open Source data available on the internet. This provides proper insights into the client's status and an ESG score. This helps the relationship managers to a great extent in making decisions.
5. Decision process: the thus collected data is processed at the final stage to make decisions on the client's proposal. The ESG data and score are used in the process of decision making. Thus, this last process ensures an excellent green finance transaction between the finance sector and the client companies. Therefore, artificial intelligence plays a crucial role in analyzing green finance for environmental development.

## 4. Proposed Work

To investigate different types of interdependence in large databases at the same time, a multiple network study is proposed. We focus on machine learning networks with four layers related to linear, nonstationary, and partial relationships among a variety of green financial time series. We use a standard network filtering blockchain process to build the sparse graph across each layer, and then we examine the finished sequencing networks (WSN with AI). The time transformation of the financial data multiplex reveals significant changes in the network's implicit multiplex properties. Other such changes are associated with times of financial stress in green finance. Some functionality deviates from the sequencing structure but is undetectable if the single-layer networks are equivalent.

For a financial related items filtered graph (FMFG) algorithm, a machine learning algorithm including one hidden unit of network output nodes *t*_*j*_ is used. The outcome of an illusionary node *i* is determined by processing the connections' *m*_*i*_ weights and its discriminatory practises descriptor *n*_*i*_^(1)^. The equation to calculate *m*_*i*_ is as following:

(1) $$\begin{array}{c} m_{i}=\overset{n}{\underset{i=1}{\sum }}g^{\left(1\right)}\left(n_{i}^{\left(1\right)}+\overset{n}{\underset{j=1}{\sum }}h\left(i,j\right)t_{j}\right). \end{array}$$

*h* in equation (1) represents a weight factor and *h*(*i*, *j*) represents the weight connecting input *j* to hidden unit *i*. Similarly, its efficiency of *f* nerve fibers is evaluated. The equation for *h*_*i*_ is given as follows:

(2) $$\begin{array}{c} h_{i}=f^{\left(2\right)}\left(n_{i}^{\left(2\right)}+\overset{n_{t}}{\underset{j=1}{\sum }}h\left(i,j\right)t_{j}\right). \end{array}$$

As such, an example *n*_*t*_ represents a hidden layers layer and *h* represents a strength linking hidden unit *j* to reduce overall  *i*. The discrimination inputs in a traditional regression model function similarly to the *h*_*i*_ is coefficient of determination. The shifts *f*^(1)^ and  *f*^(2)^ of equation (2) enable the network to form nonlinear relationships between the data. The following defines the sigmoid function, which is an example of a commonly used frequency response.

(3) $$\begin{array}{c} f\left(d\right)=\frac{1}{1+\text{finance}\left(-d\right){\;}^{\prime }}, \end{array}$$

(4) $$\begin{array}{c} f\left(d\right)=\overset{d-1}{\underset{d+1}{\sum }}\frac{\text{expexp }\left(d\right)\;-\text{exp}\left(-d\right)}{\text{expexp }\left(d\right)\;+\text{exp}\left(-d\right){\;}^{\prime }}. \end{array}$$

The sensational integral is expressed by equation (4). In addition to the linear transfer function *f*(*d*)=*d*, a network's training *d* and *h* are critical parameters that must be approximated as part of the training procedure, which is typically based on gradient descent learning to minimize somewhat of an error signal more than just a set of labeled training analyzes.

The distinction between it and a traditional FMFG network is also that measurements have been replaced by operators, and the initial parameters have been replaced by operators. The following equation depicts a classification problem with a single data increased neuronal class represented by the hidden neurons and also the various activation location classes.

(5) $$\begin{array}{c} if\;d_{1}\text{is}\rho_{1}^{\left(1\right)}\text{and }d_{2}\text{ is}\rho_{1}^{\left(2\right)}\text{ then class}=gln1. \end{array}$$

In which *ρ*_1_^(1)^ and *ρ*_1_^(2)^ are the descriptions for *d*_1_ and *d*_2_, respectively. All relationships with much the same lingual label must be associated with the same Nefclass. It is chosen to share the *gln*1 by the rule components *d*_1_ but also *d*_2 _, so its description the same in both systems.

Assume researchers have such a data set *d* of *U* different factors {(*d*_*i*_, *h*_*i*_)_*i*=1_^*U*^} to insert data  *d*_*i*_ ∈ *H*^*n*^ as well as goal directions *g*_*i*_ ∈ {0,1} for an m-class classification problem. With each specified input *d*_*i*_, *h*_*i*_ sets *h*_*i*_ sets *d*_1_^(*i*)^,…,  *d*_*n*_*i*__^(*i*)^. The regulation and oversight classifier then involves the following steps outlined below.

**Step 1 .**

Choose another sequence from  *H*, (*d*_*j*_, *g*_*j*_).

**Step 2 .**

Multiplications *d*_*i*_, *i*=1,…, *n* for each processing element to find the classifier  *d*_*l*_*i*__^(*i*)^ stipulated in the following equation:

(6) $$\begin{array}{c} \;d_{l_{i}}^{\left(i\right)}\left(d_{i}\right)=\left\{d_{l}^{\left(i\right)}\left(d_{i}\right)\right\}. \end{array}$$

If there is no concept node *Q*, the computation is as shown in the following equation: .

(7) $$\begin{array}{c} Q\left(d_{1},H\right)=\;d_{l_{i}}^{\left(i\right)},\ldots ,x\left(d_{n},H\right)=d_{l_{n}}^{\left(n\right)} \end{array}$$

If *g*_*j*_(*d*)=1, build a network to *x*(*x*_*i*_, *Q*), which is the mass including both input  *d*_*i*_ and doctrine node *H*, but it also interacts with production class node *d*. Continue until all of the constructions in *H* have been closely investigated.

The sigmoid function equation, as well as the CNN algorithm, can also be used to train various co stream neural networks. The neural network algorithm's steps must be followed.

(1) In the first place, the following equation is used to determine the outcomes among all neural networks.
  (8) $$\begin{array}{c} \text{netFre}h_{j}=\sum_{i}d_{ij}n_{i}. \end{array}$$
(2) As within the following equations , the mean-squared results of expected and actual deliverables are compared.
  (9) $$\begin{array}{c} n_{j}\left(t\right)=f\left({\text{netfreh}}_{j\;}\right)+\sum_{i}d_{ij}n_{i}, \end{array}$$
  (10) $$\begin{array}{c} F1=\frac{1}{2}\sum_{j}{\left(g_{j-}{\hat{g}}_{j}\right)}^{2}+\;\overset{t}{\underset{j=1}{\sum }}n_{j}\left(t\right). \end{array}$$
(3) Backward: the transfer function (gradient) of a mistake to excess fat is calculated for every network, as can be seen in the following equation:
  (11) $$\begin{array}{c} \frac{n_{j}\left(t\right)}{d_{ij}n_{i}}=\frac{\sum_{j=1}^{t}n_{j}\left(t\right)}{f\left({\text{netfreh}}_{j\;}\right)}.\frac{f\left({\text{netfreh}}_{j\;}\right)}{d_{ij}n_{i}}=d_{j}\left(t\right).O_{i}. \end{array}$$
(4) The iterative equation as in the following equation is being used to notify network parameters.
  (12) $$\begin{array}{c} d_{ij}\left(t+1\right)=\overset{j}{\underset{i=1}{\sum }}d_{ij}\left(t\right)+\overset{i}{\underset{j=1}{\sum }}d_{j}\left(t\right).O_{i}\left(t\right). \end{array}$$

This is decided whether it should iterate further or to stop. By associating also every neuron from a map with an input vector in overall, the briefest length measure {‖*x* − *d*_*ij*_‖} could be ascertained as in the following equation:

(13) $$\begin{array}{c} \left\{\left\|x-d_{ij}\right\|\right\}=\overset{j=1}{\underset{i=1}{\sum }}n\;\left\{\left\|d>-d_{ij}\right\|\right\}. \end{array}$$

*d*_*ij*_ is the *x* in this equation, and though  *d*_*ij* _ seems to be the *i*th neuron in the graph. The strength training has changed since establishing the neural network, and also the achievement neuron or its neighbours have already been connected to  *x*. The following equations are being used to measure the confirmation of the ith node and its neighbours, as can be seen in the following equation:

(14) $$\begin{array}{c} d_{ij}\left(tn+1\right)=\sum d_{ij}\left(tn\right)+\overset{g}{\underset{i=1}{\sum }}\alpha \left(tn\right)t_{gi}\left(tn\right)\left[d\left(tn\right)-d_{ij}\left(tn\right)\right]. \end{array}$$

*tn* denotes the step size; notwithstanding,  *t*_*gi*_ denotes the residential area kernel among unit *i* and the neural network component  *g*, that is described as both a *α*, *σ* linear transformation within the following equation:

(15) $$\begin{array}{c} t_{gi}\left(tn\right)=\left(tn\right).exp\left(\overset{n}{\underset{i=1}{\sum }}\frac{\sqrt{H_{gi}^{2}}}{\sqrt{2\sigma_{t}^{2}}}\right). \end{array}$$

Through equation (15), *H* measures the length between units *i* and also the hidden layers, but also *σ*^2^ denotes the neighborhood perimeter at  *t*. The training time decreases during this procedure, and also the neighborhood *H*_*gi*_^2^ radius seems to be the same duration as schemed just at the start.

The linearization difference is calculated using the measured, at which *n* represents the number of eigen values but also mc represents the correlating *n*. The compression ratio evaluates the *d*_*ij*_ and *H*_*ij*_ applicability of a neural network graph to information by determining the average length now between neural networks, because each data variable is analyzed.

(16) $$\begin{array}{c} H_{ij}=\frac{1}{n}\overset{n}{\underset{i=1}{\sum }}\left\|t-d_{ij}\left(tn+1\right)\right\|. \end{array}$$

The geographical imprecision, as described in that other equation here, is the fraction of measurement techniques with first and minute which are not comparable to one another, as defined in the following equation:

(17) $$\begin{array}{c} N_{ij}=\frac{1}{n}N\left(t\right)+\overset{n}{\underset{i=1}{\sum }}d\left(tn\right)-d_{ij}\left(tn\right). \end{array}$$

**Figure 2:**
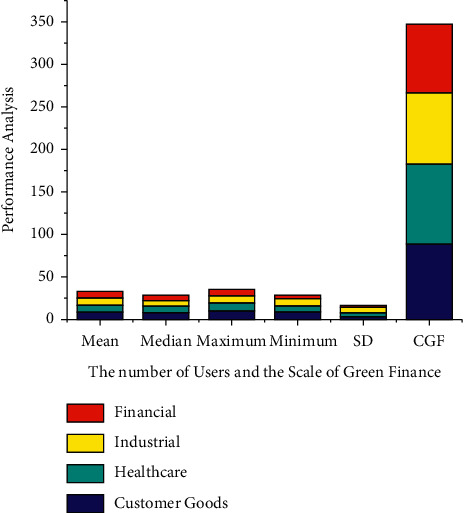
The increase in the number of users and also the scale of green finances.

If neural networks seem to be contiguous, *N*_*ij*_ equals 1, else it equals 0 and is given in the following equation:

There is also no universal definition of green marketing. Green finance is defined as financial support for green development that significantly reduces pollution-depleting substances and air poison discharges. Green development is defined as customer goods development that occurs as a result of cooperation between its economy (financial) and environment. Green finance is investing in modern but also financial advancements that reduce ozone-harming material emissions as well as other ecological microbial contamination. Green development seems to be the solution to three current threats to the global economy: environmental transformation, energy imperatives, and a monetary emergency for healthcare. Green finance represents some far experiment of each government's customary financial law *h* denotes a weight factor, but *h*(*i*, *j*) denotes the weight that connects input *j* to hidden neuron *i*. Similarly, the effectiveness of *f* nerve fibers is assessed based on this to retriever on Figure 2. The increase in the number of users and also the scale of green finances are based on evaluating the mean, median, maximum, minimum, and standard deviation (refer Table 1) green finance management classification.

**Figure 3:**
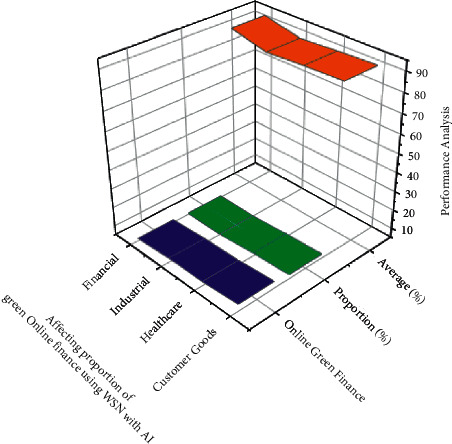
Performance analysis for affecting proportion of green online finance using WSN with AI.

In the *f*^(1)^ and  *f*^(2)^ shifts allow the network to establish nonlinear relationships among data. The sigmoid function can be defined by equation (3) based on obtaining the result in Figure 3. The customer goods a larger role in the profession of private equity, influencing and shaping the hierarchy schedules able to represent willing loan options. The green fund contains the transformation of natural corruption locations of the industry, such as air pollution, water contamination and scarcity, stream encroachment absolutely inappropriate for transmission of mechanical, medicinal, and healthcare squandering, deforestation, and loss of green space and biodiversity. It must have been environmental friendly and contributed to poverty alleviation. It is a critical way to integrate the monetary component of the transition to reduced but also asset-efficient economies, as well as to adapt to changes in the environment. Performance result analysis for affecting proportion of green online finance variables using WSN with AI financial management (refer Table 2).

In Figure 3 green finance would be a broad term that refers to money invested in viable advancement endeavors and activities, customer goods, healthcare, and industrial and financial agreements that enable the development of a more manageable economy. The online green finance includes, but is not limited to, the environment fund. It also alluded to a broader range of other natural goals, such as modern contamination control, water sanitation, or biodiversity insurance. Moderation, but also adjustment back, is particularly associated with environmental change related exercises: alleviation, monetary streams make reference to investments in ventures and initiatives that contribute to lowering or keeping a strategic distance from ozone-depleting substances discharges, while adjustment, financial streams make reference to speculations which also contribute to reducing the vulnerability of persons and goods to the effects of global climate change. Performance result analysis for affecting proportion of green online finance (refer table variables using WSN with AI proportion of the online financial management.

**Figure 4:**
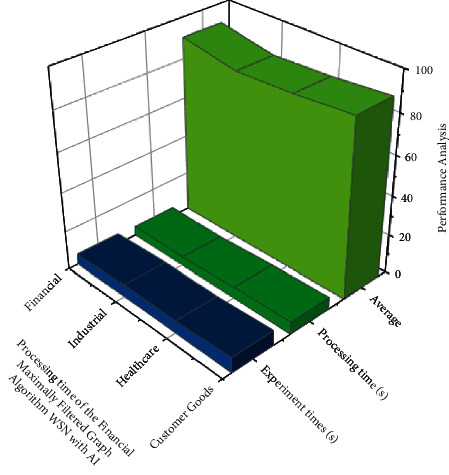
Performance analysis for processing time of the financial maximally filtered graph algorithm WSN with AI.

In Figure 4 customer goods, healthcare, industrial and financial benefits of technological advancement, the global economy is being subverted by three biggest challenges: environmental transition, which prevents the possibility, and a financial emergency. This seems to be due to the fact that financial progress communicates alongside itself based on experimental time. Costs to countries in the form of environmental destruction green finance is gaining popularity. The solution to the problem of achieving a contract between the economy and nature finance for the environment is regarded as a form of financial assistance for green development that reduces ozone depletion. The exterminating depleting stimulant discharges of processing time (refer Table 3) as well as wind pollution emissions Green funding for gardening, green structures, green security, as well as other green activities should have been prioritized. For increasing the country's monetary development an effort has been to investigate the published research on green finance. The *d*_*gi* _ denotes the length among both units *i* as well as the hidden layers in equation (15), but *σ*^2^ also denotes the neighborhood perimeter at *t*. The learning time decreases throughout that procedure, and the neighborhood radius appears to be shown in Figure 4.

**Figure 5:**
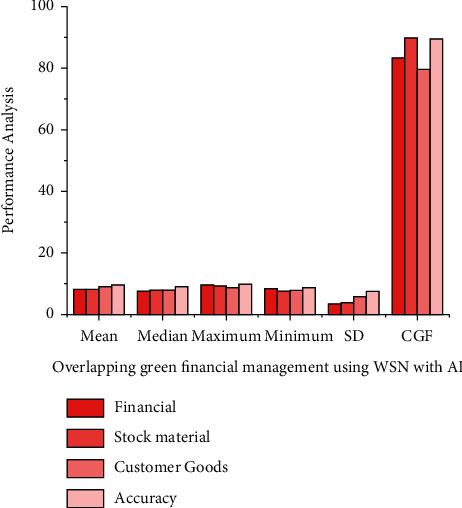
Analysis correlation of overlapping green financial management using WSN with AI.

In Figure 5, green manufacturing has yet to establish itself as such a financially feasible option, owing to the market's abundance of lower-cost alternatives. Unlike whatever is happening in Europe, in which the market for green financial products and services is expanding rapidly, the international market is still in its early stages, with no defined limits and no unified qualities that distinguish it from industrial jobs. Financial firms, particularly banks, have such an important role to play in contributing to the development of a strong as well as successful reduced economy that they must increase the use of sustainability reports in lending activities and financial investments. Analysis and correlation of overlapping green financial management using WSN with AI is done to evaluate the mean, median, standard deviation, maximum, minimum, correlation green finance of the customer goods for the analysis in stack materials and financial calculate the accuracy (refer Table 4). The regularization distance is calculated and used in the measurement results, where *n* denotes the number of eigen vectors and mc denotes the corresponding *n*. Since each information variable is analyzed, the compression evaluates the *d*_*ij*_ and *H*_*ij*_ potential application of a neural network. Figure 5 showsinformation besides finding the average length already between neural networks.

The initiative will assist them in proactively considering the environment while also creating long-term value for their business. Enterprises with a higher environmental impact will be viewed as risky financially in the future, and banks may refrain from order to finance such business owners in favor of financing technological solutions that capture or reduce emissions. This evaluation indicates that different actors have put in a lot of effort to gain traction in terms of incentivizing and measuring green finance. It demonstrates that green financial flows through financial institutions can be roughly estimated. However, it also emphasizes the need for extra efforts to produce green financial products that are more responsible and recognizable.  Table 5 provides the comparison results for the existing method. It provides the training (90.34%), testing evaluation (94.23%), and overall accuracy (95.54%). In our proposed method we provide the training analysis for (92.58%), the testing analysis for (95.34%), and overall accuracy (98.85%) to provide best result in our proposed method.

## 5. Conclusion

Green finance is a procedure or a model that is designed to allocate financial resources for the development of the environment. The allocation can be provided for the maintenance of natural resources, ecology, and human development, and in some cases, it also focuses on the reduction of human intervention in natural disasters or calamites. It can also be allocated for the social well-being of the environment where the person lives. In this research, the allocation of green finance resources is considered to be shared among transportation, healthcare needs, industrial sectors, and the persons in need of financial management. The allocation of resources is analyzed based on the online request submission and the time taken for processing the request. For this analysis, this research has implemented the financial maximally filtered graph (FMFG) algorithm and compared it with the existing neural network model. The results show that the proposed model outperforms the existing algorithm by obtaining 3.31% higher accuracy than the existing model. The proposed algorithm is compared with the neural model and it is observed that the proposed model has obtained 98.85% of accuracy, which is higher than the neural model. In the future, work can be enhanced with intelligent systems to detect and involve all processes of resource allocation systems.

## Figures and Tables

**Table 1:** Green financial management classification results.

Parameter	Customer goods	Healthcare	Industrial	Financial
Mean	8.16	8.12	8.83	7.38
Median	7.38	7.85	7.17	5.93
Maximum	9.49	9.17	8.98	7.66
Minimum	8.35	7.33	7.94	4.53
Standard deviation	3.38	3.68	6.85	2.36
Correlation of green finance	88.25	94.59	83.62	80.58

**Table 2:** Performance result analysis for affecting proportion of green online finance variables using WSN with AI.

Green finance management	Online green finance	Proportion (%)	Average (%)
Customer goods	8.72	7.23	90.23
Healthcare	7.28	6.64	89.45
Industrial	7.42	5.37	88.23
Financial	6.36	5.73	92.45

**Table 3:** Processing time of the algorithm.

Green finance management	Experiment times (s)	Processing time (s)	Average (%)
Customer goods	7.76	6.23	89.23
Healthcare	6.23	5.67	86.45
Industrial	6.46	4.35	85.23
Financial	5.34	4.78	90.45

**Table 4:** Correlation of overlapping green financial management.

Parameter	Financial	Stock material	Customer goods	Accuracy
Mean	8.18	8.17	8.82	9.36
Median	7.39	7.89	7.72	8.92
Maximum	9.42	9.12	8.73	9.63
Minimum	8.38	7.34	7.64	8.59
Standard deviation	3.36	3.67	5.74	7.38
Correlation green finance	83.29	89.59	79.47	89.54

**Table 5:** Comparison result analysis for green finance management.

Algorithm	Training (%)	Testing (%)	Accuracy (%)
Financial maximally filtered graph (FMFG) algorithm	92.58	95.34	98.85
Existing method: neural network algorithm	90.34	94.23	95.54

## Data Availability

The datasets used and/or analyzed during the current study are available from the corresponding author on reasonable request.
